# ADHD at the workplace: ADHD symptoms, diagnostic status, and work-related functioning

**DOI:** 10.1007/s00702-021-02309-z

**Published:** 2021-02-02

**Authors:** Anselm B. M. Fuermaier, Lara Tucha, Marah Butzbach, Matthias Weisbrod, Steffen Aschenbrenner, Oliver Tucha

**Affiliations:** 1 Department of Clinical and Developmental Neuropsychology, Faculty of Behavioural and Social Sciences, University of Groningen, Grote Kruisstraat 2/1, 9712 TS Groningen, The Netherlands; 2 Department of Psychiatry and Psychotherapy, University Medical Center Rostock, Rostock, Germany; 3 Department of Psychiatry and Psychotherapy, SRH Clinic Karlsbad-Langensteinbach, Karlsbad-Langensteinbach, Germany; 4 Department of General Psychiatry, Center of Psychosocial Medicine, University of Heidelberg, Heidelberg, Germany; 5 Department of Clinical Psychology and Neuropsychology, SRH Clinic Karlsbad-Langensteinbach, Karlsbad-Langensteinbach, Germany; 6 Department of Psychology, Maynooth University, National University of Ireland, Maynooth, Ireland

**Keywords:** ADHD, Work, Occupational functioning, Daily functioning, Real life functioning

## Abstract

Adults diagnosed with attention-deficit/hyperactivity disorder (ADHD) commonly experience impairments in multiple domains of daily living. Work has a central role in daily life and is susceptible to ADHD due to its cognitive demands. The present study seeks to examine the nature of work-related problems and impairments of adults with ADHD, and explores the association to ADHD symptoms and neuropsychological test performance. A community sample of 1231 individuals took part in this study and completed a set of questionnaires assessing ADHD symptoms and work-related problems. Furthermore, a clinical sample of 134 adults diagnosed with ADHD were recruited from an ADHD outpatient clinic, who completed the same set of questionnaires. A subsample of 51 patients with ADHD additionally performed a neuropsychological assessment using tests of attention and executive functions. Work-related problems were found both in individuals of the community sample with symptoms of ADHD and individuals diagnosed with ADHD. Individuals with ADHD reported work related problems particularly in not meeting their own standards and perceived potential, yet it less commonly manifests in negative performance evaluations at work or job loss. ADHD symptoms, in particular symptoms of inattention, were found to be strongly associated with work-related problems, whereas neuropsychological test performance was no meaningful predictor of functioning at work. This study emphasizes the susceptibility of individuals’ functioning at work to ADHD symptoms and impairments associated with ADHD. ADHD related difficulties at work should be considered in the clinical evaluation and targeted screening at the work place to provide support when indicated.

## Introduction

Attention-deficit/hyperactivity disorder (ADHD) is a neurodevelopmental childhood disorder that persists into adulthood in a sizable number of patients and affects about 1.2–7.3% of adults world-wide (American Psychiatric Association 2013; Fayyad et al. 2007). The disorder is clinically defined by core symptoms of inattention, hyperactivity, and impulsivity, which have been associated with negative outcomes at several domains of everyday functioning (Barkley 2006; Kooij et al. 2019). Impairments in educational achievement and occupational performance seem to be likely in ADHD due to the relatively high cognitive demands of many aspects of education and at the work place (Barkley and Murphy 2010). Work is an important activity of daily living and deserves particular attention in this context, as it contributes to mental health, increases status, social integration, and economic independence (Anker et al. 2019; Ross and Mirowsky 1995). Cross-sectional and longitudinal research revealed that work performance is indeed impaired in individuals with ADHD as compared to typically developing individuals. For example, it has been shown that individuals with ADHD perform more poorly at work and quit (200% increase in risk), or have been fired from their job (66% increase in risk) more often than typically developing individuals (Murphy and Barkley 1996; Kessler et al. 2005). Moreover, several studies showed a greater risk for individuals with ADHD to be without employment and having a lower income than people without ADHD (Bangma et al. 2019; Biederman and Faraone 2006; Fredriksen et al. 2014). Furthermore, longitudinal research demonstrated that ADHD in adolescence is associated with lower work performance more than 10 years later in adulthood (Brook et al. 2013). Impaired work performance in ADHD has also been documented in college students with ADHD by comparing scores on a work performance rating scale between students with ADHD and those not having ADHD (Shifrin et al. 2010). In a consensus report, Adamou et al. (2013) noted that adults with ADHD experience impairments in all aspects related to employment, ranging from the initial job search, the interview and in employment itself.

Inconsistent findings were reported in research investigating the role of ADHD symptoms for work performance, while Anker et al. (2019) as well as Shifrin et al. (2010) failed to show meaningful associations between ADHD symptoms and work performance, Fredriksen et al. (2014) as well as Barkley et al. (2006) did find a link between ADHD symptoms and work performance**.** Some of the inconsistencies across studies may be explained by differences in methodology, as not all studies used sensitive and clinically proven measures of work performance, but instead defined work status as a categorical variable of currently being employed or not.

The present study addresses the issue of work-related problems in adults with ADHD by employing a fine-grained rating scale that was specifically developed to detect functional impairments in adults with ADHD and that is assumed to detect also minor performance decrements in this population (CADDRA 2017). This study aims to disentangle the role of ADHD symptoms for work-related problems by examining a large community sample (*n* > 1200) with no established diagnoses of ADHD as well as a clinical sample of individuals diagnosed with ADHD from an ADHD outpatient clinic. In the clinical sample of patients with ADHD, work-related problems will not only be linked to ADHD symptoms but also to cognitive abilities as assessed with neuropsychological performance tests. The outcome of an objective cognitive performance assessment seems to be particular interesting because of the cognitive requirements that many tasks at work require. We expect significant and meaningful associations between ADHD symptoms and work-related impairments both in the community sample and patients with ADHD (Fredriksen et al. 2014; Barkley et al. 2006), with stronger associations and more pronounced impairments in the clinical sample. Furthermore, we expect that at least some of the cognitive performance indicators of the neuropsychological assessment are significantly associated with work-related functioning. However, these associations may be weak because of the differences in measurement levels in both types of assessment (Barkley and Fischer 2011; Barkley and Murphy 2010; Fuermaier et al. 2015; Toplak et al. 2013).

## Methods

### Participants

#### Community sample

A community sample of 1231 participants was recruited for the purpose of this study, which included a national online platform of panel members. This platform invites people to register as a panel member and take part in online research in exchange for a financial reward. Participation for the remaining individuals was voluntary and unpaid. Participants were selected to obtain a sample evenly divided in gender and in an age ranging from 18 to 65 years. None of the individuals in the community sample reported to have been diagnosed with ADHD. Sample characteristics, including its subsamples, are presented in Table 1.

**Table 1:** Community sample characteristics and work status

	Community sample, total sample, (*n* = 1231)	Community sample, ADHD index ≥ M + 1SD (*n* = 66)
Age (years)	45.3 ± 14.3	43.4 ± 13.6
Gender (% female)	52.4	51.5
Education (years)	12.5 ± 2.5	12.4 ± 2.2
Civil status^a^ (%)	23.0/20.6/47.1/7.2/2.1	19.7/22.7/51.5/6.1/0
Living situation^b^ (%)	22.5/55.0/22.5	21.0/64.5/14.5
Work status^c^ (%)	73.1/4.9/4.0/17.9	73.0/0/3.2/23.8
CAARS-S:L—Inattention	6.7 ± 5.3	18.2 ± 5.7
CAARS-S:L—Hyperactivity	7.7 ± 5.5	18.0 ± 6.1
CAARS-S:L—Impulsivity	7.0 ± 5.6	19.3 ± 6.0
CAARS-S:L—ADHD Index	6.6 ± 5.2	19.8 ± 3.8
WFIRS—Work (mean score)	0.34 ± 0.51	1.28 ± 0.86
Item 1 (% ≥ 2)	5.8	37.1
Item 2 (% ≥ 2)	7.4	45.0
Item 3 (% ≥ 2)	7.6	52.8
Item 4 (% ≥ 2)	7.1	52.8
Item 5 (% ≥ 2)	5.1	48.0
Item 6 (% ≥ 2)	5.8	39.3
Item 7 (% ≥ 2)	5.2	39.7
Item 8 (% ≥ 2)	5.2	37.0
Item 9 (% ≥ 2)	5.1	43.6
Item 10 (% ≥ 2)	9.3	48.2
Item 11 (% ≥ 2)	4.8	51.0

*CAARS* Conners’ Adult ADHD Rating Scale, *WFIRS* Weiss Functional Impairment Rating Scale

^a^Single/in partnership/married/divorced/widowed

^b^Alone/with one more person/with more than one more person

^c^In paid work/in training or education/pensioned/unemployed

##### Community sample (total sample)

The total sample was used to estimate the prevalence rates of work-related problems and ADHD symptoms in the community.

##### Community sample (ADHD symptoms)

A subsample of the community sample was selected with elevated levels of ADHD symptoms. The CAARS *ADHD index* was employed for the selection of participants, i.e., to identify those with a score equal or higher than one standard deviation above the mean (T score ≥ 60; *n* = 66; Table 1).

##### Community sample (community comparison group)

A comparison group to patients with ADHD was selected from the community sample which roughly matches the ADHD group (total patient sample; *n* = 134) in age, gender, and education years. For each patient with ADHD, two individuals from the community sample were selected with the same or similar characteristics in the three variables of interest (age, gender, education years), yielding a community comparison group of 268 individuals. The community comparison group did not differ significantly from patients with ADHD in age, gender or education (Table 2).

**Table 2:** Characteristics of individuals diagnosed with ADHD (total patient sample, *n* = 134) and the community comparison group (*n* = 268)

	Individuals with ADHD (*n* = 134)	Community comparison group (*n* = 268)	*t*/*χ*^*2*^ (*df*)	*p*
Age (years)	34.1 ± 11.7	34.2 ± 11.3	− 0.082 (399)	0.935
Gender (% female)	35.1	39.0	0.493 (1)	0.483
Education (years)	12.2 ± 2.3	12.5 ± 2.4	− 1.046 (399)	0.296
ADHD symptom presentation^a^	51/2/67/14	–	–	–
Comorbidity^b^	62/24/9/8/2/2/2/1/1/1	–	–	–
Stimulant drug treatment (%yes)	10.4	–	–	–
Work status^c^	55.3/27.3/1.5/15.9	73.9/13.6/0/12.5	18.229 (3)	< 0.001*
CAARS-S:L—Inattention	22.3 ± 6.7	7.5 ± 6.0	21.561 (399)	< 0.001*
CAARS-S:L—Hyperactivity	18.3 ± 7.4	8.8 ± 6.2	12.737 (399)	< 0.001*
CAARS-S:L – Impulsivity	19.7 ± 7.9	7.9 ± 6.8	14.698 (399)	< 0.001*
CAARS-S:L – ADHD Index	21.7 ± 6.2	7.5 ± 5.9	21.967 (399)	< 0.001*
WFIRS – Work (mean score)	1.24 ± 0.68	0.50 ± 0.62	10.258 (374)	< 0.001*
Item 1 (% ≥ 2)	42.1	8.7	58.000 (1)	< 0.001*
Item 2 (% ≥ 2)	55.0	11.7	79.195 (1)	< 0.001*
Item 3 (% ≥ 2)	26.3	12.3	10.792 (1)	0.001*
Item 4 (% ≥ 2)	31.1	13.2	15.091 (1)	< 0.001*
Item 5 (% ≥ 2)	20.0	11.4	4.325 (1)	0.038
Item 6 (% ≥ 2)	30.8	11.5	20.014 (1)	< 0.001*
Item 7 (% ≥ 2)	23.2	9.3	12.751 (1)	< 0.001*
Item 8 (% ≥ 2)	27.8	7.8	25.577 (1)	< 0.001*
Item 9 (% ≥ 2)	40.7	9.7	48.627 (1)	< 0.001*
Item 10 (% ≥ 2)	69.2	16.6	99.759 (1)	< 0.001*
Item 11 (% ≥ 2)	22.5	9.4	10.586 (1)	0.001*

*CAARS* Conners’ Adult ADHD Rating Scale, *WFIRS* Weiss Functional Impairment Rating Scale

*Significant at *p* < 0.01

^a^Inattentive presentation/hyperactive-impulsive presentation/combined presentation/not reported

^b^Number of patients affected with one or more comorbid psychiatric disorders: Mood disorder/anxiety disorder/personality disorder/substance abuse/psychotic disorder/somatoform disorder/autism/eating disorder/sleep disorder/low intelligence

^c^In paid work/in training or education/pensioned/unemployed

#### Patients with ADHD

One hundred and thirty-four individuals diagnosed with ADHD were selected for participation in this study. All individuals were referred by general practitioners, neurologists, psychiatrists, or self-referred for a diagnostic assessment to the ADHD outpatient clinic of the Department of Psychiatry and Psychotherapy, SRH Hospital Karlsbad-Langensteinbach, Germany. All individuals underwent a comprehensive diagnostic assessment by trained psychologists or psychiatrists. The diagnosis of ADHD was established based on the criteria as outlined in the Diagnostic and Statistical Manual of Mental Disorders, 5th Edition (DSM–5; American Psychiatric Association, 2013). The assessment procedure included a semi-structured interview to evaluate ADHD psychopathology (i.e., the Wender–Reimherr Interview; Rösler et al. 2008). Furthermore, a number of self-report scales were completed by all participants to quantify the retrospective and current ADHD symptom severity and psychopathology. Diagnostic veracity was corroborated by the identification of objective evidence of impairment (e.g., financial problems, failure in academic setting, risk behavior, drug use, etc.) and the consult of collateral information (e.g., employer evaluation, partner or parent-reports) whenever possible. The characteristics of patients with ADHD, including clinical information such as symptom presentation, comorbidity, and medication status, are presented in Table 2.

##### Subsample of patients with ADHD (administering neuropsychological tests)

A subsample of the group of patients with ADHD (*n* = 51) underwent cognitive testing using a neuropsychological battery. This subsample had a mean age of 36.2 years (11.4 years SD), contained 21 females, and averaged to 12.2 years of education (2.3 years SD). Most of the patients with ADHD from this sample were diagnosed with the combined (*n* = 28) or inattentive (*n* = 21) symptom presentation. One patient was diagnosed with hyperactive-impulsive presentation, while the symptom presentation was not reported for another patient.

### Materials

#### Questionnaire on individual characteristics

A questionnaire was composed for the purpose of this study asking for individual characteristics such as age, gender, educational attainment, civil status, living situation, and work status. Clinical information from patients with ADHD were obtained from clinical records, the clinical interview, as well as self-report questionnaires.

#### Conners’ Adult ADHD Rating Scale (CAARS)

The Conners’ Adult ADHD Rating Scale (CAARS; self-report; long version; Conners et al. 1998) is a 66-item inventory that addresses self-reported ADHD symptoms. Answers are scored on a four-point scale (0 = *not at all, never*; 1 = *just a* *little, once in a while*; 2 = *pretty much, often*; 3 = *very much, very frequently*). Scores on individual items are summed up yielding eight different scales, with some items contributing to more than one scale. In the present study scales scores for *Inattention*, *Hyperactivity*, *Impulsivity*, and the *ADHD index*, were used.

#### Weiss Functional Impairment Rating Scale (WFIRS)

The Weiss Functional Impairment Rating Scale (WFIRS) is a self-report measure for impairments that commonly occur in patients with ADHD and that are likely to represent the patients' targets of treatment (CADDRA, 2017). The WFIRS comprises 70 items that are divided into seven domains: *Family* (8 items), *Work* (11 items), *School* (11 items), *Life Skills* (12 items), *Self-concept* (5 items), *Social* (9 items), and *Risk* (14 items). Each item is scored on a four-point Likert scale scored from 0 to 3 (0 = *never, not at all*; 1 = *sometimes, somewhat*; 2 = *often, much*; 3 = *very often, very much*). An additional answering option is given with *Not Applicable*. A scale score per domain is calculated by summing up the responses to all items per domain (response values 0–3), and dividing this sum by the number of endorsed items (thereby not considering items that are answered with *Not Applicable*). Furthermore, any item score ≥ 2 was scored to indicate impaired functioning (CADDRA 2017)*.* The WFIRS was reported to have high internal consistency with Cronbach’s alpha > 0.8 for each domain and the scale as a whole. For the purpose of the present study, only the *Work* subscale will be considered, including both individual item scores (scores ≥ 2) and the *Work* mean score. Table 3 presents the eleven items comprising the *Work* subscale of the WFIRS.

**Table 3:** WFIRS *Work* subscale

Item number	Item
#1	Problems performing required duties
#2	Problems with getting your work done efficiently
#3	Problems with your supervisor
#4	Problems keeping a job
#5	Getting fired from work
#6	Problems working in a team
#7	Problems with your attendance
#8	Problems with being late
#9	Problems taking on new tasks
#10	Problems working to your potential
#11	Poor performance evaluations

*WFIRS* Weiss Functional Impairment Rating Scale

Answers options include 0 (never), 1 (sometimes), 2 (often), 3 (very often), or ‘not applicable’

#### Neuropsychological performance tests (subsample of patients with ADHD)

A number of neuropsychological tests were performed to assess aspects of attention and executive control, i.e., selective attention (Perception and Attention Functions—Selective attention, WAFS), vigilance (Perception and Attention Functions—Vigilance, WAFV), cognitive flexibility (Trail Making Test, TMT), verbal fluency (Regensburg Word Fluency, RWT), working memory (N-back Verbal, NBV), and planning (Tower of London—Freiburg version, TOL-F). The tests WAFS, WAFV, TMT, NBV, and TOL-F were taken from test battery Cognitive Functions ADHD (CFADHD; Tucha et al. 2013; Schuhfried 2010). The CFADHD is a computerized test battery assessing cognitive functions in which adults with ADHD commonly experience difficulties. The RWT is a test administered via paper-and-pencil (Aschenbrenner et al. 2000). The administration of the total test battery took about 60 min. Test variables assessing speed (mean reaction time), variability (SD of reaction time), and accuracy (e.g., errors of omissions, commissions, or correct responses) were derived (see Table 6 for on overview of all test variables derived from this battery).

### Procedure

#### Community sample

Participants of the community sample took part in the study online. All participants provided active informed consent by clicking the option in the online form that they agreed with participation in this study. Participants were first requested to complete the questionnaire asking for personal characteristics, followed by the CAARS and WFIRS, taking about 15–20 min in total. Only participants that completed the survey were considered for data analysis. The study was approved by the Ethical Committee Psychology (ECP) affiliated with the University of Groningen, The Netherlands.

#### Patients with ADHD

Patients with ADHD gave written informed consent and completed the survey as well as the test battery, if applicable, as part of a larger research project. Participation in this project was voluntary, unpaid, and was separated from their diagnostic assessment and treatment. All patients with ADHD were asked to complete the set of questionnaires to the best of their knowledge and not to seek help from the examiner or to discuss questions or their responses. A subsample of patients with ADHD (*n* = 51) additionally performed the neuropsychological battery using cognitive tests. Tests were administered in an office of the psychiatric hospital without distraction. Test administration was led by a trained psychologist and took about 60 min. The study involving patients was approved by the medical ethical committee affiliated to the University of Heidelberg, Germany.

### Statistical analysis

Data were presented by group in descriptive statistics, i.e., giving mean scores and standard deviations (for continuous data) and frequencies (for categorical data). Responses of patients with ADHD and the community comparison group were compared using t-tests for independent samples (continuous data) and Chi-Square tests (categorical data). Associations between ADHD symptom severity and WFIRS *Work* scores were explored in bivariate correlation analyses. The association between neuropsychological test performance of the subsample of patients with ADHD and work-related problems was explored in multiple linear regression analysis with the neuropsychological test scores as predictors and the WFIRS *Work* score as the criterion. Furthermore, the association between each of the test variables and the WFIRS *Work* score was examined in bivariate correlation analyses. Significance level was adjusted to 0.01 to control for alpha error inflation in multiple testing.

## Results

**Fig. 1:**
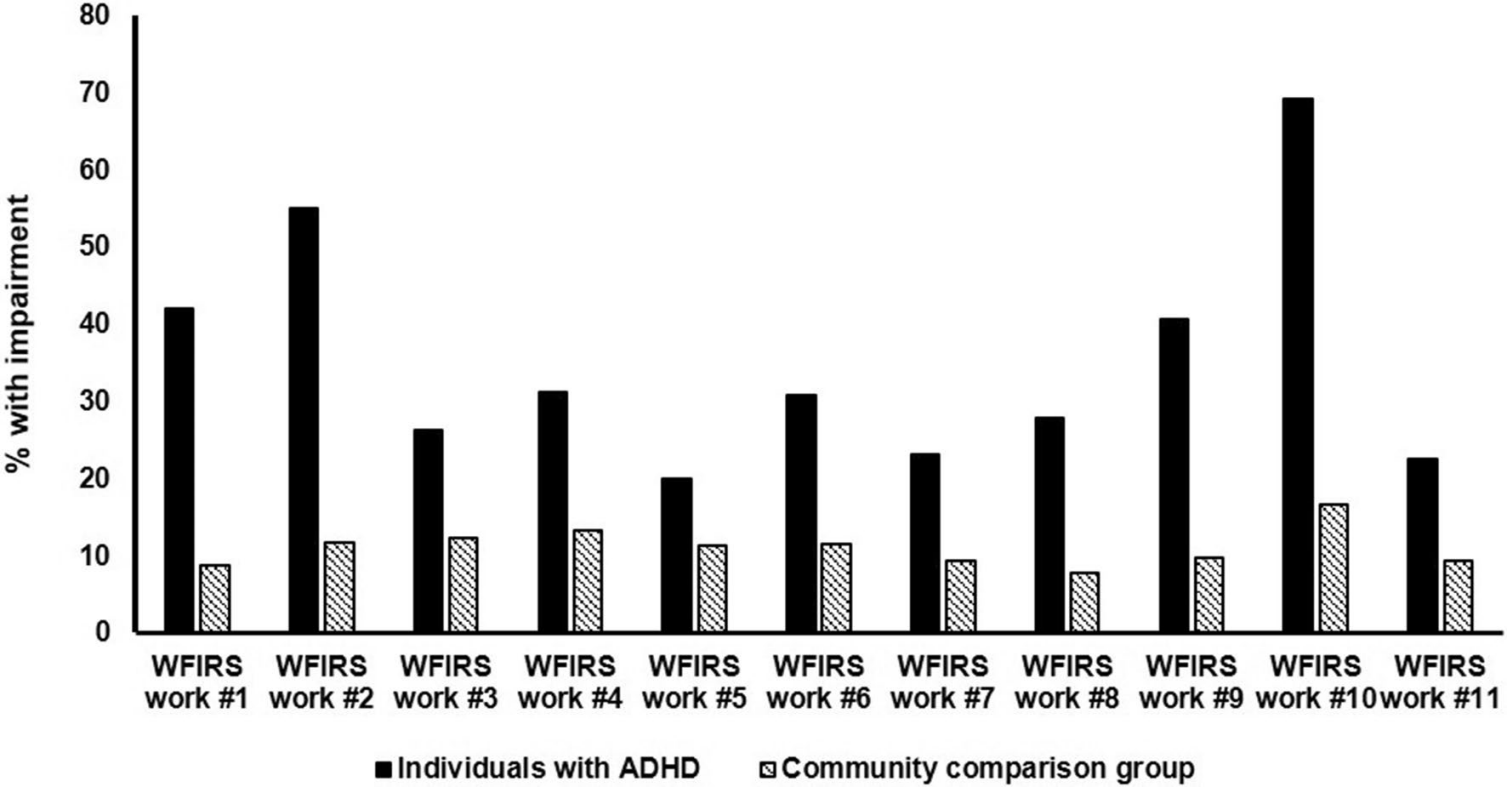
Comparison of the proportion of individuals indicating impairment (score ≥ 2) in each of the WFIRS *Work* item between patients with ADHD (*n* = 134) and the community comparison group (*n* = 268)

ADHD symptom scores per domain, work status, the WFIRS *Work* score, and impairments in each of the WFIRS *Work* items per group are presented in Tables 1 and 2. The majority of the individuals of the community sample (about 73%) and both of its subsamples were currently in paid work. Whereas about 5–9% of the community sample reported to experience impairments in each of the *Work* items, a considerable larger proportion of impairments (in 37 to 53% of participants) were observed in the subgroup of the community sample with elevated levels of ADHD symptoms. A larger variation in work-related problems was observed in individuals diagnosed with ADHD, with 20 to 23% of patients with ADHD reporting impairments in items #5 (“getting fired from work”), #7 (“problems with your attendance”), and #11 (“poor performance evaluations”), whereas up to 55 to 69% of patients with ADHD reported impairments in items #2 (“problems with getting your work done efficiently”) and #10 (“problems working to your potential”). Group comparisons revealed that patients with ADHD reported significantly more often work-related problems than the community comparison group in each of the *Work* items, with the exception of item #5. Differences between patients with ADHD and the community comparison group are visually depicted in Fig. 1. The cumulative percentages of work-related problems are presented in Table 4, which contrasts the ADHD group with the community comparison group. Whereas more than 80% of the community sample reported to have no or one work-related problem, it is more than 80% of patients with ADHD who have at least one problem, and in most cases (69% of patients with ADHD) even more than one. Also, it is shown that a smaller proportion of individuals of the ADHD group were currently in paid work (55%) compared to the community samples, with more individuals being in training or following education (27%).

**Table 4:** Cumulative percentages of the number of *Work* impairments of patients with ADHD (*n* = 134) and the community comparison group (*n* = 268)

Number of *Work* impairments	Patients with ADHD (*n* = 134)	Community comparison group (*n* = 268)
0	19.4	72.4
1	31.3	82.8
2	44.8	85.1
3	59.7	88.4
4	70.1	90.7
5	79.1	91.8
6	84.3	92.9
7	90.3	94.0
8	93.3	96.3
9	96.3	97.4
10	99.3	98.9
11	100	100

Correlation analyses demonstrated mostly moderate to strong associations between the WFIRS *Work* score and ADHD symptoms in all four groups examined, with largest associations found to inattention symptoms (Table 5). Associations between the WFIRS *Work* score and symptoms of hyperactivity did not reach significance in both the community sample with elevated levels of ADHD symptoms and individuals diagnosed with ADHD.

**Table 5:** Pearson correlation analyses (r, p) between WFIRS *Work* score and ADHD symptoms in different subsets of the community sample and patients with ADHD

	WFIRS *Work* score			
	Community sample, total sample, (*n* = 1231)	Community sample, ADHD index ≥ M + 1SD (*n* = 66)	Community sample, comparison group (*n* = 268)	Individuals with ADHD (*n* = 134)
CAARS-S:L—Inattention	0.596, < 0.001*	0.467, < 0.001*	0.577, < 0.001*	0.397, < 0.001*
CAARS-S:L—Hyperactivity	0.415, < 0.001*	0.310, 0.014	0.437, < 0.001*	0.229, 0.011
CAARS-S:L—Impulsivity	0.519, < 0.001*	0.375, 0.003*	0.534, < 0.001*	0.345, < 0.001*
CAARS-S:L—ADHD Index	0.569, < 0.001*	0.294, 0.020	0.575, < 0.001*	0.462, < 0.001*

*CAARS* Conners’ Adult ADHD Rating Scale, *WFIRS* Weiss Functional Impairment Rating Scale

*Significant at *p* < 0.01

Table 6 presents neuropsychological test performance of the subsample of patients with ADHD, together with bivariate correlations to WFIRS *Work* scores that depict the association between each test variable and work-related problems. Test results revealed cognitive impairments in about one third to almost half of the patients with ADHD in aspects of selective attention (reaction time, variability of reaction time, and omissions), vigilance (omissions and commissions), flexibility (TMT-B), and verbal fluency, whereas functioning in working memory and planning was impaired in a fewer number of patients (around a quarter of patients). None of the test variables revealed significant associations to the WFIRS *Work* score. Furthermore, regression analysis on this subsample failed to find a significant model that predicts WFIRS *Work* scores when entering all neuropsychological test scores into one model; *F*(13,32) = 0.481; *p* = 0.920; *R*^2^ = 0.163.

**Table 6:** Cognitive performance scores and bivariate associations to WFIRS *Work* score in a subsample of patients with ADHD (*n* = 51)

Cognitive functions with test variables	Min	Max	Mean	SD	% PR ≤ 16	Correlation (*r*) to WFIRS Work *
Selective attention						
WAFS—RT (ms)	215	600	404	95	36.0	0.08
WAFS—SD	1.10	1.48	1.26	0.09	39.2	< 0.01
WAFS—Omissions	0	10	1.16	2.04	37.3	0.13
WAFS—Commissions	0	26	4.57	4.96	29.4	0.01
Vigilance						
WAFV—RT (ms)	229	656	441	83	14.0	0.21
WAFV—SD	1.14	3.48	1.30	0.33	25.5	0.17
WAFV—Omissions	0	20	2.14	3.66	37.3	0.09
WAFV—Commissions	0	27	3.20	4.95	33.3	0.10
Cognitive flexibility						
TMT-A—Time (s)	11.2	40.6	18.6	5.2	24.0	0.15
TMT-B—Time (s)	13.2	82.8	34.0	13.5	32.0	0.03
Verbal fluency						
RWT—Produced words	7	26	15.96	4.46	44	– 0.01
Working memory						
NBV—Correct responses	3	15	11.14	2.99	24.5	– 0.11
Planning						
TOL-F—Planning score	6	20	14.81	3.07	22.9	– 0.10

*WAFS* perception and attention functions—selective attention, *WAFV* perception and attention functions—vigilance, *TMT* trail making test, *RWT* regensburg word fluency test, *NBV* N-back Verbal, *TOL-F* tower of London—Freiburger Version

^*^ None of the correlations met statistical significance level *p* < 0.01 (all *p*’s ≥ 0.148)

## Discussion

Initial analyses on work-related functioning were performed in a large community sample of individuals equal or older than 18 years, no established diagnoses of ADHD, and who were in paid work at the time of the assessment in 73% of the cases. In this sample, we observed only a small number of self-reported work-related problems, with rates of 5–9% per item. Work-related problems in the community appeared to be clearly associated to ADHD symptoms, which is evidenced by high rates (37 to 53%) of work-related problems and a heightened WFIRS *Work* score in the subsample of the community with elevated ADHD symptoms (CAARS *ADHD index* at least one standard deviation above the mean). Furthermore, correlation analyses underlined the role of ADHD symptoms in work-related functioning by showing moderate to strong associations between ADHD symptom severity and functioning at work in both the total sample of the community as well as the subsample of individuals with elevated ADHD symptoms. Comparing the roles of different ADHD symptom domains, it becomes apparent that symptoms of inattention may have the strongest predictive ability for functioning at work.

The sample of individuals diagnosed with ADHD was less often in paid work at the time of the assessment compared to the community (55% vs. 73%), as more patients with ADHD were currently in training or followed an education (27% vs. 5%). Compared to the community comparison group, patients with ADHD reported marked impairments in several work-related aspects of functioning, with significantly higher scores in all but one (#5; “*getting fired from work*”) item. High scores are observed in particular in “*getting your work done efficiently*” (#2; 55%) and “*working to your potential*” (#10; 69%). Fewer impairments were reported on “*getting fired from work*” (#5; 20%), “*problems with your attendance*” (#7; 23%), and “*poor performance evaluations*” (#11; 23%). This discrepancy in the occurrence of work-related problems indicate that patients with ADHD commonly feel that their ADHD hinders their functioning at work and performing to their perceived potentials, and results in not meeting their own standards, but still their work performance may not always strike out negatively to their managers (as seen in performance evaluations) and may not result in losing their jobs in most cases. Further, the group comparisons showed that problems of adults with ADHD at work do not only refer to work performance itself, but also to situations involving social interaction with colleagues and supervisors (#3 *“problems with your supervisor”*; #6 *“problems working in a team”*). The number of impairments across all eleven aspects of functioning of the WFIRS *Work* scale (Table 4) further demonstrates the impact of work-related problems in individuals with ADHD. Whereas the majority of the community comparison group indicate no (72.4%) or up to one (82.8%) impairment, more than half of the patients with ADHD report at least three impairments, and more than 20% report at least six work-related impairments. Also in the group of patients with ADHD, work-related problems (WFIRS *Work* score) was significantly associated with ADHD symptoms, even though of slightly smaller size than in the community sample, with correlation coefficients reaching medium size for symptoms of inattention and impulsivity, and non-significant correlations of small size for symptoms of hyperactivity. The significant and meaningful associations between work-related problems and ADHD symptoms in all groups of this study is in agreement with results of Fredriksen et al. (2014) as well as Barkley et al. (2006), but was not found in the study of Anker et al. (2019) who reported no significant associations between work status and ADHD symptoms and Shifrin et al. (2010) who also failed to show associations between ADHD symptoms and work performance in students with ADHD. In this context, it must be noted that the applied methodology of assessing work performance differed across studies, as, for example, Anker et al. (2019) did not use a scale for the assessment of work performance, but defined a dichotomous ‘work’ variable depending on whether individuals reported work as the main source of income. A dichotomous work status variable may have restricted the variance and concealed differences The WFIRS *Work* scale as used in this study is a more fine-grained measure and is presumably more sensitive to detect work-related problems and limitations in this population compared to a dichotomous variable of having work or not.

Neuropsychological performance on tests for attention and executive control was shown, against our expectations, to have no predictive value for work-related problems in adults with ADHD. A lack of meaningful association between neuropsychological test performance and occupational functioning has already been demonstrated in earlier work (Barkley and Murphy 2010; Barkley and Fischer 2011), which showed that self-ratings of executive functions outperformed test performance in their predictive value for occupational functioning in individuals with ADHD. This differential utility of self-ratings vs. test performance for the prediction of functioning in major life activities has been explained by differences in measurement levels of both types of assessments (Barkley and Murphy 2011; Toplak et al. 2013; Fuermaier et al. 2015). The characteristics of both types of assessment might be best described by the distinction between optimal performance (psychometric tests), and typical performance (self-ratings). Psychometric tests are believed to assess the efficiency of cognitive processes (optimal performance), whereas, in contrast, self-ratings assess the extent to which individuals achieve their goals in typical day-to-day situations and thereby provide an indication of individuals’ goal pursuit (typical performance; Toplak et al. 2013). The better predictive value of (self-rated) ADHD symptoms over neuropsychological test performance for work-related problems may support this assumption.

It is concluded that work-related issues are common in individuals diagnosed with ADHD but also in individuals with symptoms of ADHD, supporting a dimensional conceptualization of ADHD (Bitto et al. 2017). Adequate attention to work-related difficulties should, therefore, be given in the clinical evaluation of ADHD and treatment planning. Furthermore, a targeted work place screening for ADHD symptoms and related impairments seems to be important to provide support when needed. For example, work place adjustments have been suggested by Adamou et al. (2013), targeting work performance that is associated with various ADHD symptom domains (Adamou et al. 2013). Given the present study revealed that in particular symptoms of inattention are strongly associated with work-related problems, it appears reasonable to assume that adjustments compensating for cognitive symptoms of inattention and disorganization may be especially beneficial, such as providing structured notes and agendas, regular supervision with feedback, breaking down targets, goals, and work units, regularly introducing change, providing a quieter room or positioning in office, arranging flexi-times, or introducing incentive and reward systems (Adamou et al. 2013). Behavioral based treatment programs that are proven to reduce ADHD symptomatology may also be helpful to improve functioning at work place. It is stressed that work place adjustments and behavioral based treatment planning need to be tailored to the type of work and its cognitive demands to unfold optimal effectiveness.

### Limitations and future directions

This study is associated with several limitations. Firstly, the group of patients with ADHD is a selected sample of individuals from an ADHD outpatient clinic, and may not represent the population of ADHD adequately. ADHD has a heterogeneous character with inter-individual differences in several demographic and clinical variables that may affect work-related functioning, such as symptom presentations (Sobanski et al. 2008), co-existence of other psychiatric disorders (Anker et al. 2018; Fredriksen et al. 2014; Sobanski et al. 2007), and stimulant drug treatment. Other factors, such as educational achievement and socioeconomic variables may also play a role for work performance in adults with ADHD (Anker et al. 2019; Fredriksen et al. 2014), which may confuse the identification of key factors and the ability to extrapolate conclusions based on a sample.

Second, information on ADHD symptoms and work-related functioning is based on self-report, which may be prone to biases commonly seen in studies using self-report measures in psychiatric samples in general (i.e., over-reporting; Dandachi-Fitzgerald et al. 2011) and in this population in particular (i.e., under-reporting; Manor et al. 2012). Future studies addressing work performance and functioning are, therefore, advised to corroborate self-reported information with other-reports or objective evidence of performance and functioning at work.

Third, while work-related issues observed in the subsample of the community with elevated levels of ADHD symptoms may seemingly be associated to ADHD symptoms, it must be considered that this selected group of the community may show general signs of psychopathology, decreased well-being, and problems in their daily life’s functioning, which may turn into, but not exclusively, high scores on scales for ADHD symptoms and work-related problems. Work-related impairments observed in this group and high scores on ADHD symptoms may co-occur, and may not be causally linked to each other.

Finally, it must be noted that this study failed to consider the diversity of jobs with their respective cognitive demands. This is important to stress as different types of work may pose different challenges to individuals with ADHD, and may result in different answers to the items used in this assessment of work-related problems. For example, endorsements to statements such as “problems performing required duties”, “problems with getting your work done efficiently”, and “problems with working to your potential” may likely depend on the characteristics and (cognitive) requirements of the specific job. The issue of occupational diversity and complexity may need to be studied in further research when trying to achieve a more accurate and balanced analysis of work-related problems experienced by individuals with ADHD.

## General conclusions

This study identified a number of work-related problems in both individuals with symptoms of ADHD and individuals diagnosed with ADHD. Individuals with ADHD experienced problems at work in particular in not meeting their own standards and perceived potential, yet this is less often accompanied by negative performance evaluations or losing their job. ADHD symptoms, in particular symptoms of inattention, were found to be strongly associated with work-related problems, whereas neuropsychological test performance did not predict work-related problems. Even though the measurement scale of this study was subjective and the design correlational in nature, the present results emphasize the susceptibility of individuals’ functioning at work to ADHD symptoms and impairments. Work-related issues should be considered in the clinical evaluation but also at the work place by means of targeted screening for ADHD symptoms and related impairments. Self-reports should preferably be complemented by other reports (e.g., partner, colleague, or employer report) and objective data of work performance and functioning.

## Data availability statement

The data that support the findings of this study are available from the corresponding author upon reasonable request.
